# Infertility and associated factors in three hospitals in Douala, Cameroon: a cross-sectional study

**DOI:** 10.4314/ahs.v20i4.57

**Published:** 2020-12

**Authors:** Thomas Obinchemti Egbe, Charmaine Ngo Mbaki, Nicholas Tendongfor, Elvis Temfack, Eugene Belley-Priso

**Affiliations:** 1 Department of Obstetrics and Gynaecology, Douala General Hospital, Cameroon; 2 Faculty of Health Sciences, University of Buea, Cameroon; 3 Mbingo Baptist Hospital, Mboppi, Douala, Cameroon; 4 Department of Clinical Research and Internal Medicine, Douala General Hospital; 5 Department of Obstetrics and Gynaecology, Faculty of Medicine and Biomedical Sciences, University of Yaoundé, Cameroon

**Keywords:** Couple infertility, prevalence, associated factors, Douala, Cameroon

## Abstract

**Aim:**

We determined the prevalence and factors associated with couple infertility in three hospitals in Douala, Cameroon.

**Methods:**

We conducted a cross-sectional study from December 18th 2015 to March 18th 2016 in three public hospitals in Douala. Three hundred and sixty participants were studied prospectively for associated factors using a multivariate logistic regression model and 4732 files were studied retrospectively for the prevalence of infertility. Statistical significance was set at p < 0.05.

**Results:**

The prevalence of couple infertility was 19.2%. In logistic models, the factors which independently increased the risk of couple infertility were a history of reproductive tract infection/STI, a history of uterine fibroids, a history of dysmenorrhea and abortion for the females while for males it was a history of mumps, erectile dysfunction and exposure to chemicals/toxic substances/pesticides.

**Conclusion:**

One in every five couples in this study was infertile. Several factors affect the risks associated with couple infertility. The identification of these factors could help detect subgroups of couples at high risk of infertility. Reproductive health education, screening programmes for STI's that may lead to infertility should be offered to couples.

## Introduction

Infertility is a disease of the reproductive system defined as the failure to achieve a clinical pregnancy after twelve months or more of regular unprotected sexual intercourse or due to an impairment of a person's capacity to reproduce, either as an individual or with his/her partner 1. It is a public health problem that affects about 8% – 12% of couples worldwide 1. Infertility is further categorized as primary or secondary. The primary infertile female is a woman who has never been diagnosed with a clinical pregnancy and meets the criteria of being classified as having infertility. Secondary female infertility applies to a woman unable to establish a clinical pregnancy but who has previously been diagnosed with a clinical pregnancy 2. Secondary infertility is the most common form of female infertility worldwide especially in regions like sub-Saharan Africa where there are high rates of unsafe abortions, poor maternity care leading to post-abortive and postpartum infections^3^, ^4^. Besides, there has been a fertility decline in Asia, Latin America and the developed world but Africa has noticed little reproductive changes during the same periods^5^. Infertility affects about 10% to 30% of couples in sub-Saharan Africa 6. Furthermore, women were responsible for 25%–37% of infertility in Africa^7^ although Sule et al., reported that female infertility accounted for 51.5% of patients admitted to the gynaecology departments of four hospitals in Osun State Nigeria, over a two-year- period 8. Also, Abubakar et al. in 2011 reported a prevalence of infertility of 15.7% in a teaching hospital in Sokoto, Nigeria 9.

More than 90% of male infertility results from low sperm count (oligospermia), poor sperm quality (teratozoospermia), or both. The factors associated with couple infertility are hypogonadotropic hypogonadism, hyperprolactinaemia, disorders of ciliary function, cystic fibrosis, infections, systemic diseases and lifestyle-related factors/diseases 10–13. Premature ovarian insufficiency, polycystic ovary syndrome, endometriosis, uterine fibroids and endometrial polyps may play a role in female infertility 12, 13. Male infertility may be due to testicular and post-testicular deficiencies 14–16. Semen decline that has been observed over the last 50 years, endocrine-disrupting chemicals and consanguinity are other factors that may be involved 17–21,22.

In Cameroon, issues relating to reproductive health are usually geared towards contraception and family planning. Policy makers have so far not taken infertility and related problems sufficiently seriously. There is paucity of data on the prevalence and associated factors of infertility in Cameroon. Moreover, the causes of infertility are multifactorial and any gender could be at the origin of infertility. However, in Cameroon, the female gender bears much of the blame for this problem. The men, on the other hand, cling to traditional practices to protect their ego and do not recognize infertility as a medical condition. Socio-cultural perceptions and beliefs among African people still predominate in our setting. Consequently, establishing the burden of infertility and identifying the associated factors is fundamental in understanding and demystifying this health problem. Infertility, unlike other medical conditions, involves both partners and several dynamic associated factors. Identifying the aetiologies and associated factors in our society will, therefore, be beneficial to health care providers in establishing a diagnosis to institute appropriate management.

Parenthood is an important achievement for married couples in our society. It is customary for the families of the couple to expect the announcement of an expected baby within less than a year of marriage. However, not all couples that desire a pregnancy will achieve one spontaneously and a proportion of the couples would need medical assistance to resolve the underlying fertility problems.

Due to the misconception and ignorance of the general population about this condition, there is need for replacement of traditional beliefs with scientific facts thus making such studies essential.

Data on infertility in our society is lacking. As such, providing data on the prevalence and associated factors of infertility in Cameroon would inform clinicians and health policy makers on the severity of this condition, its public health implications and the need for investment of resources in its management. We hypothesize that the burden of couple infertility in Douala is high and that there are several associated factors, especially of infectious origin. This study aimed to determine the prevalence and associated factors of infertility in three hospitals in Douala, Cameroon.

## Materials and methods

### Study design and site

We conducted a hospital-based cross-sectional study from December 18^th^ 2015 to March 18^th^ 2016 where data was collected prospectively from participants who came to the OPD of the three public hospitals in Douala. Simultaneously, we studied files of those who consulted at the obstetrics and gynaecology unit of the three hospitals from 2011 to 2015 for the prevalence study. A total of 4732 files were studied, among which 858 had the diagnoses of infertility.

Douala is a metropolitan city and the economic capital of Cameroon with about 3 million inhabitants. The three hospitals studied included a tertiary care centre (the Douala General Hospital, DGH), a secondary care centre (the Douala Laquintinie Hospital DLH) and a primary care centre (the Bonassama District Hospital BDH).

### Study population

#### Accessible population

The target population included couples attending the Obstetrics and Gynaecology units either for infertility related complaints or for obstetrical reasons.

### Inclusion criteria

We enrolled consenting females of reproductive age (15–49 years) and their partners who consulted for infertility related issues during the study period. We also included consenting couples consulting for pregnancy follow up (antenatal care) during the study period, to compare their characteristics with those who were infertile.

### Exclusion criteria

We excluded women with other gynaecological problems and those who were not in the reproductive age group. We also excluded women who had tried to conceive for less than one year, those who conceived (with difficulty) after infertility or medical treatment, those receiving any form of contraception and women with a history of hysterectomy.

### Sampling

#### Sampling method and sample size calculation

Sample sizes were determined for the 95% confidence interval with a design effect of 1.1 using the Lorenz formula^23^. A hospital-based study carried out by Aubakar et al. 2011 in Sokoto Northwest Nigeria reported that the prevalence of infertility was 15.7% 9. With this assumption, a sample size of 203 participants would be required. With a projected subject dropout rate of 10% the total number of subjects required for study was 223. Participants were selected consecutively as they reported daily for consultation.

### Study procedure

#### Participant enrolment and data collection

For the prospective phase of the study and after obtaining ethical clearance from the Institutional Ethics Committee for Research on Humans of the University of Douala (Ref. No IEC-UD/462/02/2016/T) and administrative approvals from the Douala General Hospital (Ref No 087AR/MINSANTE/HGD/DM/01/16), the Douala Laquintinie Hospital (Ref. No 48/AR/MINSANTE/DHL/CM), and the Bonassama District Hospital (Ref. No 931/AR/MSP/DRSTLT/SSDB/HDB), participants who came for Obstetrics and Gynaecology consultation were approached at the reception before or after their consultation with the attending physician. The study objectives and procedure were explained to them. Interested participants signed the consent form and were enrolled for study. A pretested structured questionnaire was administered to eligible participants. Information collected from participants was: socio-demographic data: age, occupation, level of education, and marital status. The obstetric history included number of pregnancies, outcome of pregnancy, puerperal infections, history of pelvic procedures and duration of trial for a pregnancy. The gynaecological history included: menstrual irregularities, history of STIs, unsafe abortion, pelvic surgeries, uterine fibroids, PCOS, endometriosis and contraceptive use. The sexual history such as frequency of coitus, multiple/new sexual partners was elicited.

The men were asked about a history of mumps, testicular torsion, varicocele, genital injury/swelling, genital surgery and genital infections.

Lifestyle assessment included: alcohol consumption, cigarette smoking, use of recreational drugs, and exposure to chemicals, toxins and radiation.

### Data management and analysis

The data were entered in Microsoft Excel 2013 and exported to the Statistical Package for the Social Sciences (SPSS) version 25 (SPSS, Inc., Chicago, IL, USA). The demographic characteristics and the frequency of reproductive tract infections were summarized using descriptive statistics and the results were displayed in tables. Associations between fertility status, obstetrical, menstrual and coital history were compared in male and female using the student t-test and the Man &Whitney test. Missing data were coded and not counted in the final analysis. A multivariate logistic regression model was fitted to identify factors associated with infertility in males and females. A p-value < 0.05 was considered statistically significant.

This study was conducted according to the Helsinki declaration^24^ and reported following the Strobe guidelines for cross-sectional studies.

## Results

During the prospective phase of the study, 370 couples were contacted, and 97.3% (360/370) accepted to participate, and 121 of the 360 participants were diagnosed with infertility.

### Prevalence of infertility

A total of 4732 files were studied in the retrospective phase of the study with 858 cases of infertility while in the prospective phase of study, there were 360 participants of whom, 121 were infertility giving a prevalence of: 858+121÷4732+360=979÷5092×100=19.23%

As shown in table 1, most study participants were in the age group 30–39 years with mean age of women being 30.1 (SD 5.7) years and that of men 36.4 (SD 7.5) years. Infertile couples were older than fertile couples, were married and their professions were in the informal sector (self-employment, business, traders etc.).

**Table 1:** Socio-demographic characteristics of study participants

Variable	Levels	Male	Female
**Age group**	**Mean age ± SD**	36.43 ± 7.5	30.1 ± 5.7
	**>40**	115 (31.9)	28 (7.8)
	**20–29**	60 (16.7)	167 (46.4)
	**30–39**	185 (51.4)	165 (45.8)

	**Total**	**360**	**360**

**Marital status**	**Cohabiting**	71 (19.7)	71 (19.7)
	**Married**	220 (61.1)	216 (60.0)
	**Separated**	4 (1.1)	4 (1.1)
	**Single**	65 (18.1)	69 (19.2)

	**Total**	**360**	**360**

**Profession**	**Formal**	145 (40.3)	98 (27.2)
	**Informal**	206 (57.2)	96 (54.4)
	**Unemployed**	9 (2.5)	66 (18.3)

	**Total**	**360**	**360**

Table 2 shows that the median menstrual cycle length in days (p=0.01), frequency of coitus/week, median (range) (p=0.001), the median number of previous pregnancies (p=0.001), the median number of previous deliveries (p=0.001) and the median number of previous abortions (p=0.001) was different between the fertile and infertile couples.

**Table 2:** Reproductive health characteristics of study population

Characteristics	Variable	Infertile couples (N = 121)	Fertile couples (N = 239)	p-value
**Characteristics** **of menstrual** **cycle**	Menarche in years, mean (SD)	13.8 (1.7)	13.7 (1.5)	0.142
	Cycle length in days, median (Range)	28 (21 – 57)	28 (21 – 37)	**0.01**
	Duration of periods in days, median (range)	4 (2 – 14)	4 (1 – 14)	0.066

**Coital history**	Age of first coitus in years, mean (SD)	18.0 (2.0)	18.4 (2.6)	0.14
	Frequency of coitus/week, median (range)	3 (1 – 7)	2 (0 – 5)	**0.001**

**Obstetrical** **history**	Previous pregnancies, median (range)	2 (0 – 6)	3 (1 – 7)	**0.001**
	Previous deliveries, median (range)	0 (0 – 5)	1 (0 – 6)	**0.001**
	Number of previous abortions, median (range)	1 (0 – 3)	0 (0 – 5)	**0.001**

**Note:** The tests used here are student's t-test for means and the Man & Whitney test for median.

The range here gives a better appraisal of the variation in the responses than the interquartile range

### Factors associated with couple infertility

As shown in table 3, male partners in the age groups 20–29 years and 30–39 years and those who had previously fathered a child were less likely to have infertility (AOR 0.09; 95% CI: 0.03–0.26, p=0.000), (AOR 0.31; 95% CI: 0.18–0.54, p=0.000) and (AOR 0.31; 95% CI: 0.17–0.57, p=0.000), respectively. Furthermore, male participants with a history of mumps (AOR 2.9; 95% CI: 1.54–5.46, p=0.001), erectile dysfunction (AOR 4.6; 95% CI: 2.1–10.2, p=0.000) and exposure to chemicals/toxic substances (pesticides) (AOR 17.4; 95% CI: 3.1–98.9, p=0.001) were more likely to be infertile.

**Table 3:** Male factors associated with infertility in couples in Douala (Multivariable analysis)

Variable	Levels	AOR	95% CI	p-value
**Age group**	30–39	0.30	0.17 – 0.53	0.000
	20–29	0.06	0.02 – 0.20	0.000
	>40	1		

**Fathered a child**	Yes	0.31	0.17 – 0.57	0.000
	No	1		

**History of mumps disease**	Yes	2.77	1.46 – 5.25	0.002
	No	1		

**Erectile dysfunction**	Yes	4.47	1.46 – 5.25	0.000
	No	1		

**Ejaculation problems**	Yes	3.5	0.35 – 36.24	0.284
	No	1		

**Exposure to chemicals/toxic** **substances/pesticides**	Yes	17.39	3.06 – 98.85	0.001
	No	1		

**Smoking**	Yes	2.65	0.92 – 7.62	0.07
	No	1	.	.

**AOR: Adjusted odd ratio, CI: Confidence interval**

**Note: 1.** Here we modeled Infertile as the response, treating fertile as the reference category. The model fitted here is a multiple logistic regression model. We have to interpret adjusted odd ratios and confidence intervals.

**2.** The two Predictors Testicular torsion and History of genital injury did not contribute anything to the model and were excluded from the model.

Table 4 shows that female participants with a history of reproductive tract infection/STI/PID (AOR 2.4; 95% CI: 1.34–4.40, p=0.004), those with a history of uterine fibroids (AOR 3.4; 95% CI: 1.65–7.09, p=0.001) those with a history of painful menses or dysmenorrhea (AOR 2.6; 95% CI: 1.34–5.18, p=0.005) and abortion (AOR 1.9; 95% CI: 0.99–3.59, p=0.05) were more likely to be infertile.

**Table 4:** Female factors associated with infertility in couples in Douala (Multivariable analysis)

Variables	Levels	AOR	95% CI	p-value
Female age group	30–39	0.172	0.06 – 0.54	0.003
	20–29	0.115	0.03 – 0.39	0.000
	>40	1		

History of sexually transmitted infection	Yes	2.483	1.37 – 4.49	0.003
	No	1		

Pelvic surgery on ovaries	Yes	0.622	0.27 – 1.44	0.268
	No	1		

Uterine fibroids	Yes	3.409	1.66 – 7.02	0.001
	No	1		

Painful menses or dysmenorrhea	Yes	2.683	1.37 – 5.26	0.004
	No	1		

Pain during intercourse or dyspareunia	Yes	1.955	0.90 – 4.23	0.089
	No	1		

Abortion	Yes	1.889	0.99 – 3.59	0.052
	No	1		

Had more than one sexual partner	Yes	1.557	0.80 – 3.04	0.194
	No	1		

**AOR: Adjusted odd ratio, CI: Confidence interval**

**Note: 1**- Here we modeled Infertile as the response, treating fertile as the reference category

**2**- The variable diagnosis or treatment of endometriosis could not enter the model because of data is dislocated

On the other hand female participants who were in the age groups (20–30 years) and (30–39 years) had a 12.5% and 18% chance of having infertility.

As shown in table 5, the most frequent microorganisms found among participants were *Chlamydia trachomatis*, *Mycoplasma homini*s and *Ureaplasma urealyticum*.

**Table 5:** Frequency of reproductive tract infections among couples seeking care for infertility in Douala

Diagnosis	Females, N (%)	Males, N (%)
**Chlamydia**	78 (50.3)	83 (53.2)
**Mycoplasma**	19 (12.3)	18 (11.5)
**Ureaplasma**	14 (9.0)	0 (0)
**Syphilis**	11 (7.1)	13 (8.3)
**Gonorrhea**	6 (3.9)	28 (18.0)
**Chlamydia and** **mycoplasma**	11 (7.1)	0 (0)
**Chlamydia and syphilis**	11 (7.1)	11 (7.1)
**Chlamydia and** **ureaplasma**	5 (3.2)	0 (0)
**Chlamydia and gonorrhea**	0 (0)	2 (1.3)
**Gonorrhea and syphilis**	0 (0)	1 (0.6)

## Discussion

This study aimed to determine the prevalence and factors associated with infertility in three hospitals in Douala, Cameroon. The prevalence of infertility was 19.2%. The factors associated with female infertility included a history of RTI, presence of uterine fibroids, dysmenorrhea and abortions, while exposure to chemicals/toxins/pesticides, a history of mumps, and erectile dysfunction was associated with male infertility.

### Prevalence of infertility

It has been previously reported that the burden of infertility is more among the low-income countries of sub-Saharan Africa and an infertility belt has been described extending from Angola to the Sudan including Cameroon, Gabon, Nigeria 25. The reported prevalence of 19.2% in this study could even be higher because cultural and social barriers impede couples from disclosing fertility problems to healthcare providers. This is consistent with other studies 9, 26, 27. The fertility rate in Cameroon has dropped from 6 births per woman in 1966 to 4.8 births per woman in 2015 28. That notwithstanding, the fertility rate for urban towns like Douala and Yaoundé are 3.2 and 3.5 births per woman, respectively^28^, ^29^. This is particularly low compared to other areas in Cameroon with high fertility rates like the Extreme North Region and West Region that have fertility rates of 6.8 and 6.0 births per woman, respectively 28, 29. However, this infertility prevalence was lower than the rates reported for other sub-Saharan countries. Sule et al., and Polis CB et al. reported a prevalence of 51.8% and 31.1% (95% CI: 27.9–34.7%) 8, 30. The difference in prevalence may be due to study setting and the definition of infertility. In our study, we used the WHO definition of infertility as inability to conceive after ≥12 months of regular, unprotected sexual intercourse in a woman of reproductive age (15–49 years) while Sule and Polis used demographic approaches that considered 5-years periods with no births though such long periods may be less clinically relevant. Also, Polis et al. used the current duration (CD) approach technique to estimate a population-level time-to-pregnancy (TTP) distribution and infertility prevalence using a cross-sectional design. This approach samples couples at risk of pregnancy at the time of interview and determines their current length of time-at-risk of pregnancy with the advantage that it is a cost-efficient cross-sectional study design, that also includes all couples at risk of pregnancy regardless of prior fertility history (e.g. childless couples who may be more likely to be infertile) 2 or pregnancy intentions (e.g. couples who may be infertile but have stopped trying) 30.

### Factors associated with couple infertility

We observed a progressive increase in the prevalence of infertility with advancing age in both males and females. In the couples, the rate of infertility almost doubled with a ten-year increase in ages. The reason for this is that majority of couples in Cameroon seek medical attention for infertility relatively late after failure from traditional medicine/spiritualist. Furthermore, the referral system of patients to appropriate specialists is not well respected. The resultant effect is that, couples waste valuable time consulting ancillary medical staff; nurses, midwives or the general medical practitioners for fear of high cost in specialized centres or distance from specialized centres 12. Some of them would even want to hide their fertility problems because of cultural beliefs 31, 32. Besides, in women, advanced age is associated with a decrease in the quality and quantity of the oocytes in the ovarian reserves, thus increasing the rate of female infertility 33, 34. In males, despite the continuous sperm production throughout life, testicular function and sperm quality deteriorate with age. In both groups, the offsprings have increased rates of genetic abnormalities and women are prone to miscarriages as a result of advanced ages 34, 35. Therefore, couples with advanced age need counseling regarding the declining fertility rates/potentials of women.

In Cameroon, the pitfalls of infertility management are the inability to diagnose the cause of infertility early. This is related to lack of technical know how in some health facilities or lack of finances on the part of the couples. This is compounded by the fact that health insurance does not cover infertility matters for the small proportion of workers who have health insurance as compared to high-income countries such as Israel, France and Belgium 36, 37. For this reason, medical practitioners are sometimes obliged to use the syndromic approach to diseases (based on association of symptoms and signs) in the management of many medical conditions including infertility in Cameroon 38.

The female factors independently associated with infertility were: dysmenorrhea or painful menses, uterine fibroids, history of reproductive tract infection/sexually transmitted infections and history of abortions.

Dysmenorrhea was found to be independently associated with infertility. This occurrence may be due to underlying gynaecological problems such as chronic PID and endometriosis. Typically, endometriosis presents with dysmenorrhea, dyspareunia, chronic pelvic pain and infertility. The association of dysmenorrhea and infertility in this study is consistent with previous reports that showed that about 25 to 50% of infertile women have endometriosis, and 30 to 50% of women with endometriosis are infertile^39^. However, the diagnosis of endometriosis may be difficult in our environment because syndromic diagnosis by using signs and symptoms may be erroneous because the clinical presentation of endometriosis is variable. The gold standard for the diagnosis of endometriosis is laparoscopy and histopathology of biopsied samples though others have recently suggested clinical diagnosis and optimal medical treatment 40. There are few centres performing laparoscopic surgery in Cameroon 41, 42. Laparoscopic surgery is practiced only at the DGH among the study hospitals, though not all the gynaecologists at the DGH are proficient in the technique.

The association between menorrhagia, dysmenorrhea, intermenstrual bleeding and infertility in this study may be because a substantial proportion of our participants had uterine fibroids 43. Multiple uterine fibroids are common among black women, and are associated with infertility 44. Besides, Cameroon is in an endemic zone for uterine fibroids therefore screening for fibroids among infertile women in Cameroon should be the mainstay^12^. Uterine leiomyoma's are present in approximately 5–10% of patients presenting with infertility, although independently associated with infertility in only 1% to 2.4% of the infertile patients 45, 46. Okogbo et al. 2011 in South West Nigeria reported that 47.7% of menstrual irregularities and 31.9% of infertility cases were due to uterine leiomyoma's 47.

Reproductive tract infection or pelvic inflammatory disease (PID) is a common cause of hospitalization in most hospitals in Cameroon and other countries in sub-Saharan Africa. There is a direct correlation or risk association between *Chlamydia trachomatis* infection, PID and tubal infertility 12, 48, 49. However, another study in Cameroon reported that *Mycoplasma* species occurred more frequently than *C. trachomatis* in their study 50.

Abortion as a risk factor for female infertility is related to secondary infertility. Besides, the practice of voluntary induced abortion is illegal in Cameroon except for therapeutic abortions where the pregnancy could compromise or jeopardize maternal or foetal outcomes. This has led to increased complications from abortions performed by unqualified medical and non-medical staff thereby increasing the maternal morbidity from post-abortion infections (endometritis), uterine synechiae, infertility and death 12, 51, 52.

The factors associated with male infertility in this study were: erectile dysfunction, exposure to chemicals/toxins, and history of mumps. Cigarette smoking affected the fertility of participants in this study though it was not statistically significant (AOR 2.65; 95% CI: 0.92–7.62, p= 0.07).

In Cameroon, male factor infertility is a very difficult problem to handle because culturally, infertility is considered a female problem and women usually take the blame for not being able to procreate 53. As long as a man has full erection he will hardly accept that he is infertile despite being diagnosed with low sperm count 53. Exposure to toxins/chemicals is an important finding in this study. Cameroon is a country that thrives more on agriculture. There are companies that grow banana in Tiko, Nyombe, Penja and Palms in Bomono and Limbe. These are all feeder localities to health facilities in Douala. The companies in these localities usually spray their plantations using small aircrafts. The local farmers who do subsistence farming also spray their farms with pesticides and/or herbicides using manual pumps. The effect of these chemicals on the fertility of this population has not been studied. However, several studies based on exposure to environmental toxins suggest a negative impact on semen quality, in terms of sperm concentration, motility, and/or morphology. These toxins may exert estrogenic and/or anti-androgenic effects, which in turn alter the hypothalamic-pituitary-gonadal axis (HPGA), induce sperm DNA damage, or cause sperm epigenetic changes 17. Besides, others have reported a 7.4% prevalence of primary infertility among farm workers exposed to pesticide 54.

In Cameroon, mumps usually occurs during childhood and people do not associate the condition with infertility. However, it has been reported that mumps orchitis is associated with asthenospermia (poor sperm motility), and infertility in 30–87% patients with bilateral mumps orchitis 55.

Most men who will readily seek medical attention in Cameroon are those with erectile dysfunction (inability to have or sustain a good quality erection) (ED) and advancing male age has been associated with ED 35. Some of these men will take to alcohol and cigarette smoking thereby complicating the condition. High levels of alcohol intake do appear to be associated with changes in semen that may affect fertility. This is consistent with studies that have reported that cigarette smoking has a dose-dependent association with endothlial disease leading to erectile dysfunction and that this effect is observed more with more than 20-pack years of exposure. Cessation of smoking improves penile tumescence and rigidity 56–58.

More so, others have reported that smoking was associated with reductions in semen quality including sperm concentration, motility and morphology 59, 60. Harlev et al in South Africa reported a well-established biological finding that smoking increases the presence of reactive oxygen species, thereby resulting in oxidative stress (OS). OS has devastating effects on sperm parameters, such as viability and morphology, and impairs sperm function, hence reducing male fertility 14. Haifa reported that 38% of the infertile males who were smokers had low levels of reproductive hormones and semen parameters compared to non-smokers 61.

## Study limitations and strengths

The participants of the interview could have had recall bias of some information that could be considered private. We did not study the link between erectile dysfunction and cigarette smoking. The results obtained from this study may not represent what obtains in other parts of Cameroon because of cultural differences. Finally, the cross-sectional nature of the study could not allow for causal inference and sampling may be biased since it is not a probability sampling.

This is one of the few studies regarding infertility and its associated factors in Cameroon. This should open the way for other studies.

## Conclusion

One in every five couples in this study is infertile. The factors associated with female infertility include a history of reproductive tract infections, presence of uterine fibroids, dysmenorrhea and abortions while exposure to chemicals/toxins or pesticides, a history of mumps, and erectile dysfunction was associated with male infertility. We recommend that reproductive health education (abortion care etc.) and screening programs for sexually transmitted infections (Chlamydia trachomatis, Mycoplasma and Neisseria gonorrhea) should be offered readily to couples by healthcare providers.
